# Efficacy and Safety of Human Intravenous Immunoglobulin 10% (Panzyga®) in Patients with Primary Immunodeficiency Diseases: a Two-Stage, Multicenter, Prospective, Open-Label Study

**DOI:** 10.1007/s10875-017-0424-4

**Published:** 2017-07-29

**Authors:** Michael Borte, Isaac R. Melamed, Grazyna Pulka, Barbara Pyringer, Alan P. Knutsen, Hans D. Ochs, Roger H. Kobayashi, Ai Lan Kobayashi, Sudhir Gupta, Magdalena Strach, William Smits, Anna Pituch-Noworolska, James N. Moy

**Affiliations:** 1 Klinik für Kinder- und Jugendmedizin, Klinikum St. Georg gGmbH, Leipzig, Germany; 2 Immunodeficiency Centre Leipzig (IDCL), Hospital St. Georg gGmbH Leipzig, Delitzscher Strasse 141, 04129 Leipzig, Germany; 3 IMMUNOe Research Center Centennial, Centennial, CO USA; 4 Klinika Alergologii Collegium Medicum, Uniwersytetu Jagiellońskiego, Kraków, Poland; 5 Clinical Research and Development Department, Octapharma Pharmazeutika Produktionsges.m.b.H, Vienna, Austria; 6 Saint Louis University, St. Louis, MO USA; 7 Department of Pediatrics, University of Washington and Seattle Children’s Research Institute, Seattle, WA USA; 8 UCLA School of Medicine, Los Angeles, CA USA; 9 Papillion, NE USA; 10 University of California, Irvine, CA USA; 11 Jagiellonian University Medical College, Kraków, Poland; 12 The Allergy and Asthma Center, Fort Wayne, IN USA; 13 University Children Hospital, Kraków, Poland; 14 Division of Pediatric Allergy/Immunology, Stroger Hospital of Cook County, Chicago, IL USA

**Keywords:** Primary immunodeficiency diseases, intravenous immunoglobulin, panzyga®, serious bacterial infections

## Abstract

**Purpose:**

To assess the efficacy and safety of panzyga® (intravenous immunoglobulin 10%) in preventing serious bacterial infections (SBIs) in patients with primary immunodeficiency diseases (PIDs), a prospective, open-label, multicenter, phase 3 study and an open-label extension study were undertaken.

**Methods:**

Initially, the study drug (infusion rate ≤0.08 mL/kg/min) was administered at intervals of 3 or 4 weeks for 12 months, followed by 3 months of panzyga® at infusion rates increasing from 0.08 to 0.14 mL/kg/min. The primary endpoint in the main study was the rate of SBIs per patient-year on treatment. Secondary outcomes included non-serious infections, work/school absence, episodes of fever, quality of life, and adverse events (AEs).

**Results:**

The main study enrolled 51 patients (35% female, mean age 26.8 years), with 21 participating in the extension study. The rate of SBIs per patient-year was 0.08 in the total population; there were four SBIs in the 4-weekly treatment group (2/30 patients) and none in the 3-weekly group (*n* = 21). Compared with 4-weekly treatment, 3-weekly treatment was associated with a higher rate of upper respiratory tract infections (RTIs), ear infections, and work/school absences, but a lower rate of lower RTIs and fever. Treatment was generally well tolerated; no AE led to treatment withdrawal or death.

**Conclusions:**

Overall, the use of panzyga® in patients with antibody-deficient PID was associated with a low rate of AEs and was effective in preventing SBIs, exceeding US FDA and European Medicines Agency recommendations for efficacy.

## Introduction

Primary immunodeficiency diseases (PIDs) comprise a heterogeneous group of disorders that have intrinsic defects involving the development and function of the immune system [1]. To date, >300 molecularly defined disorders have been identified with new PIDs still being added and classification ongoing [2]. Children and adults with PID and predominant antibody deficiency have an increased risk of severe bacterial, viral, and fungal infections, and present with infections that typically involve the upper and lower respiratory tracts, the gastrointestinal system, skin, and other organs [3]. Furthermore, patients with PID are at greater risk of developing malignancies [4] and autoimmune disorders [5]. Because most of these antibody deficiencies cannot be cured, affected patients require lifelong infusions with intravenous or subcutaneous immunoglobulin G (IVIG or SCIG) [6]. Replacement therapy with IVIG or SCIG provides patients with predominant antibody deficiency with specific antibodies, thus preventing serious bacterial and viral infections and reducing the number and duration of hospitalizations, as well as the loss of school/work days [6, 7].

This report describes the results of a phase 3 study of 12-month duration and its 3-month extension; the main study objective was to assess efficacy and safety of two treatment schedules of 10% IVIG (panzyga®; Octapharma AG, Lachen, Switzerland) in preventing serious bacterial infections (SBIs), while the extension study provided data on the tolerability of panzyga® administered at high infusion rates.

## Methods

The protocols for both studies were reviewed and approved by each study site’s Independent Ethics Committee or Institutional Review Board before the study commenced. The studies were conducted in accordance with the ethical principles of the Declaration of Helsinki and the International Conference on Harmonization guideline E6: Good Clinical Practice. Adult patients provided written informed consent; for minors, both written informed assent (as applicable for the study site) and consent from the patient and the patient’s parent/legal guardian, respectively, were required.

### Study Design

#### Main Study

This prospective, open-label, non-controlled, non-randomized, multicenter phase 3 study examined two panzyga® infusion regimens administered every 3 or 4 weeks for 12 months in patients with PID from the USA and Europe (ClinicalTrials.gov record NCT01012323).

Inclusion criteria were age 2–75 years, confirmed diagnosis of common variable immunodeficiency disorders (CVIDs) or X-linked agammaglobulinemia (XLA), previous treatment with a commercial IVIG at a dose of 200–800 mg/kg body weight every 21–28 days for at least six infusion intervals, and evidence of an IgG trough level of ≥550 mg/dL at the previous two infusions before enrolment. Female patients of childbearing potential had to have a negative pregnancy test and use a reliable contraceptive method during the study. A minimum weight requirement was based on the blood test volumes needed for the study.

The main exclusion criteria were (1) requirement for routine premedication for IVIG infusion, (2) severe impairment of liver function, (3) abnormal renal function, (4) congestive heart failure or uncontrolled arterial hypertension, (5) a positive screening test for HIV and/or hepatitis B or C infection, (6) treatment with immunosuppressive or immunomodulatory drugs, and (7) pregnant or nursing women.

This study was designed in accordance with the US Food and Drug Administration (FDA) and European Medicines Agency (EMA) guidelines on the clinical investigation of human IVIG [8, 9].

#### Extension Study

Patients in the USA who had completed the main study and were 6 years or older were eligible to enroll in the extension study (ClinicalTrials.gov record NCT01313507). A further inclusion criterion was administration of the maximum infusion rate (0.08 mL/kg/min; 480 mg/kg/h) for the last three infusions of the main study, without need for premedication. Exclusion criteria were any condition or circumstance that would result in exclusion from the main study, administration of any immunoglobulin apart from panzyga® between the conclusion of the main study and start of the extension study, and any deviation in the patient’s treatment interval of >7 days between the last infusion in the main study and the first infusion in the extension study.

### Study Medication

Panzyga® is a ready-to-use, sterile, glycine-stabilized 10% liquid preparation of polyvalent human immunoglobulin G (IgG) for intravenous administration with physiologic osmolality (240–310 mosmol/kg). Virus safety is achieved through a combination of various process steps, including S/D treatment, ion-exchange chromatography, and nanofiltration (20 nm).

#### Main Study

Each enrolled patient received 200–800 mg/kg body weight of study drug every 21 (±3) or 28 (±3) days for 12 months, unless medical conditions or other circumstances resulted in the patient’s withdrawal from the study. Individual treatment doses and intervals were dependent upon the patient’s previous IVIG dose and dosing frequency before entry into the study. Patients received treatment using an infusion pump at the following rates: 0.01 mL/kg/min for the first 30 min, followed by 0.02 mL/kg/min for the second 30 min; infusion rates were then increased every 30 min using predefined patterns with maximum rates of 0.04 mL/kg/min (first and second infusion), 0.06 mL/kg/min (third and fourth infusion), 0.07 mL/kg/min (fifth and sixth infusion), and 0.08 mL/kg/min (all subsequent infusions). Rate increases were only made if the lower infusion rate was tolerated. Patients receiving treatment every 3 weeks had a total of 17 infusions, and those receiving treatment every 4 weeks had a total of 13 infusions.

#### Extension Study

The dose and infusion schedule remained unchanged from the main study. Patients received panzyga® using an infusion pump at the following rates: 0.01 mL/kg/min for the first 30 min, followed by 0.03 mL/kg/min for 15 min; infusion rates were then increased every 15 min using predefined patterns with maximum rates of 0.10 mL/kg/min (first infusion), 0.12 mL/kg/min (second infusion), and 0.14 mL/kg/min (all subsequent infusions). Rate increases were only made if the prior infusion rate was tolerated. Patients receiving treatment every 3 weeks had a total of five infusions, and those receiving treatment every 4 weeks had a total of four infusions.

### Treatment Outcomes

#### Main Study

The primary efficacy endpoint was the rate of SBIs (defined as bacteremia/sepsis, bacterial meningitis, osteomyelitis/septic arthritis, bacterial pneumonia, and visceral abscess) per patient-year on treatment. Secondary endpoints included the number of episodes per patient-year of other infections; the type, severity, and time to resolution of other infections; number of days of use of antibiotics per patient-year on treatment and type and dose; number of days of absence from school or work per patient-year on treatment; hospitalizations due to infection and number of days of hospitalization per patient-year on treatment; reason for hospitalization; and number of episodes of fever per patient-year on treatment. These data were collected from patient diaries which were checked by the investigator at each visit.

Safety assessments included type and frequency of adverse events (AEs), laboratory parameters (hematology, biochemistry, direct Coombs test, urinalysis, and viral markers), vital signs, and physical examination. The severity of AEs was described as mild (no significant discomfort to patient or change in routine activities), moderate (limitation in activity with possible need for some assistance; no or minimal medical intervention/therapy required), or severe (marked limitation in activity with required assistance; medical intervention/therapy required). AEs were identified as serious (SAEs) if they resulted in death or persistent or significant disability/incapacity, were life-threatening, required hospitalization or prolongation of existing hospitalization, or other important medical event.

#### Extension Study

The primary endpoint was occurrence of AEs causally and/or temporally related to panzyga® given at infusion rates of up to 0.14 mL/kg/min. The safety parameters assessed were the same as those in the main study.

### Statistical Analysis

The statistical software package used in both studies was SAS, version 9.1 or higher.

#### Main Study

The full analysis set (FAS) included all patients who received ≥1 complete treatment and had available data on infections from ≥1 post-treatment diary. The per-protocol (PP) set consisted of those patients in the FAS with no major protocol violations. The safety set included all patients who had received at least one infusion. The rate of SBI/year for each patient was presented as point estimates of the rate along with a 99% confidence interval (CI) and was calculated as *r* = (total number of SBIs) / (patient-years on panzyga® treatment). The null hypothesis was to be rejected if the upper one-sided confidence limit for SBI rate per patient-year was less than 1.0, tested at the 1% significance level. The planned sample size was 50 patients, based on an SBI frequency of <0.5/year in patients receiving regular IVIG [8], and accounting for an overall dropout rate of 15%.

#### Extension Study

The planned number of patients for the extension study was 20–35, based on the number of patients enrolled in the main study from US study sites who completed the study at the maximum infusion rate and without the need for premedication and did not meet the exclusion criteria for the extension study.

## Results

### Patient Characteristics

#### Main Study

**Fig. 1:**
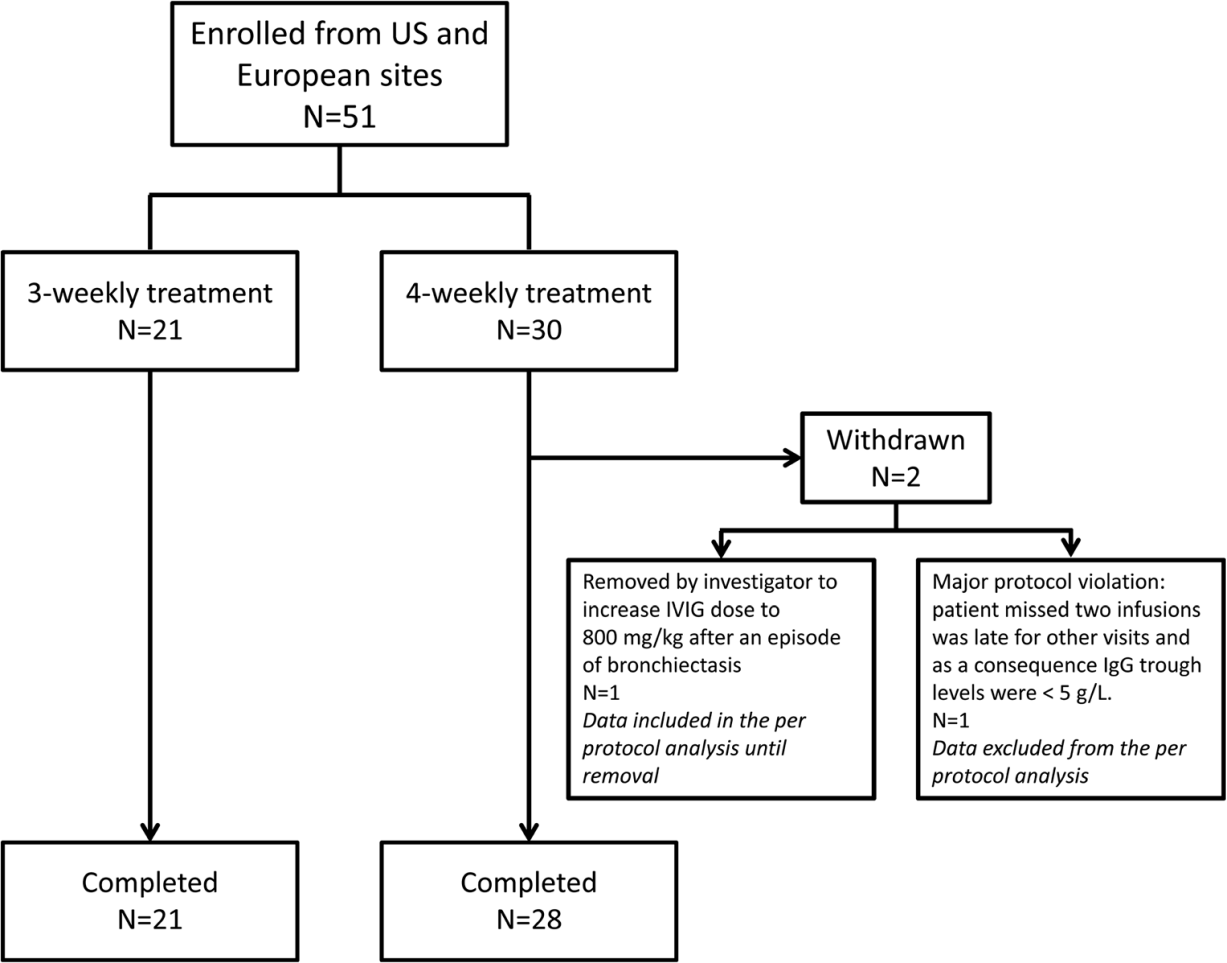
Patient disposition through the main study

In total, 51 patients (13 children [≥2 to <12 years], 12 adolescents [≥12 to <16], and 26 adults [≥16 to ≤75]) were enrolled from 11 study centers, seven in the USA and four in Europe. The patient disposition through the study is outlined in Fig. 1. One patient with bronchiectasis on a 4-week schedule was removed from the study after nine infusions at the investigator’s discretion, in order to increase the IVIG dose to 800 mg/kg following exacerbation of the lung disease. All patients enrolled in the study received at least nine infusions and provided data on infections by at least nine post-treatment diaries, so all 51 patients were included in the FAS. One patient on the 4-week infusion schedule was excluded from the PP set for major protocol violations, including missing two infusion visits. The remaining patients (*n* = 50) comprised the PP analysis set.

There were no substantial differences in baseline characteristics between the two treatment groups (Table 1). Briefly, the mean age of the total population was 26.8 years (range 2–65 years), 35% were female; 14% of all patients were of Hispanic or Latino ethnicity. Twenty-one patients (41%) were treated with 3-weekly infusions, and 30 patients (59%) with 4-weekly infusions (Table 1). Of the 51 patients enrolled in the study, 43 patients (84.3%) were diagnosed with CVID and eight patients (15.7%) with XLA.

**Table 1:** Baseline characteristics and demographics of the main study population

Parameter	3-weekly IVIG schedule (*n* = 21)	4-weekly IVIG schedule (*n* = 30)	Total (*n* = 51)
Gender, *n* (%)			
Female	7 (33.3)	11 (36.7)	18 (35.3)
Male	14 (66.7)	19 (63.3)	33 (64.7)
Age (years)			
Mean ± SD	26.2 ± 21.2	27.2 ± 18.2	26.8 ± 19.3
Min, max	2, 65	5, 63	2, 65
Race, *n* (%)			
White	21 (100.0)	30 (100.0)	51 (100.0)
Ethnicity, *n* (%)			
Hispanic or Latino	3 (14.3)	4 (13.3)	7 (13.7)
Not Hispanic or Latino	18 (85.7)	25 (83.3)	43 (84.3)
Not reported	0 (0.0)	1 (3.3)	1 (2.0)
Height (cm)			
Mean ± SD	156.3 ± 25.3	156.7 ± 23.6	156.5 ± 24.1
Min, max	90, 191	108, 186	90, 191
Weight (kg)			
Mean ± SD	59.7 ± 31.4	57.9 ± 23.0	58.7 ± 26.5
Min, max	13, 145	18, 100	13, 145
BMI (kg/m^2^)			
Mean ± SD	22.8 ± 8.6	22.3 ± 4.6	22.5 ± 6.5
Min, max	15, 52	15, 32	15, 52

*BMI* body mass index, *IVIG* intravenous immunoglobulin, *SD* standard deviation

The mean duration of treatment was 360 days. Overall, there were 740 infusions with an actual mean IgG dose of 417 g (range 104–1224 g) per patient, and a mean dose per infusion of 485 mg/kg body weight. The mean duration of each infusion was 2.2 h (range 1.4–4.3 h). Of the high-rate infusions, 90.1% were administered at the maximum rate of 0.08 mL/kg/min.

#### Extension Study

The extension study enrolled 21 patients (eight children [age ≥ 6 years to <12], three adolescents [≥12 to <16 years], and ten adults [≥16 to ≤75]) from six US centers. All patients completed the study. Thirty-eight percent of patients were female and the mean age of the patient cohort was 23.8 years (range 6–62 years). Hispanic or Latino ethnicity was reported by 19% of patients. Most patients (76.2%) had CVID, while the remainder had XLA.

The mean treatment duration was 107 days. Patients had a total of 96 infusions. The actual mean dose of IgG per infusion was 29.8 g or 542 mg/kg body weight. The mean duration of each infusion was 1.5 h (range 1.2–2.0 h), and 85.2% of the high rate infusions were administered at the maximum rate of 0.14 mL/kg/min.

### Efficacy

#### Primary Endpoint

In the FAS, all SBIs reported were bacterial pneumonia, and occurred in patients receiving 4-weekly infusions (Table 2). The rate of SBIs per patient-year was less than 1.0 in all treatment groups (Table 2). This result was confirmed in the PP set, from which one patient with an SBI was excluded, with a rate of SBI per patient-year for all patients of 0.061 (99% CI 0.0060–0.6246); of note, the excluded patient who experienced an SBI missed two infusion visits and was late for others and had low IgG levels during the study (≤500 mg/dL).

**Table 2:** Serious bacterial infections (SBIs) per patient-year in the main study population

SBI	3-weekly IVIG schedule (*n* = 21)	4-weekly IVIG schedule (*n* = 30)	Total (*n* = 51)
Total no. of SBI, *n*	0	4	4
Bacterial pneumonia	0	4	4
Total no. of patients with SBI, *n* (%)	0 (0.0)	2 (6.7)	2 (3.9)
Bacterial pneumonia	0 (0.0)	2 (6.7)	2 (3.9)
Number of patient years exposure	20.5	29.7	50.2
Rate of SBI per patient-year	na	0.135	0.080
One sided 99% CI, upper limit	na	0.849	0.503

*CI* confidence interval, *IVIG* intravenous immunoglobulin, *na* not applicable, *no.* number

#### Secondary Endpoints

For both treatment schedules, trough levels of serum IgG were almost constant throughout the study. Patients on a 4-week infusion schedule had median IgG trough levels of 810–870 mg/dL, while patients on a 3-week schedule had median levels of 1100–1220 mg/dL. Over 75% of patients reported a non-serious infection (Table 3). Infections occurring in >25% of the total population included lower (LRTI) and upper respiratory tract infections (URTIs) and infections of the gastrointestinal tract. Patients in the 3-weekly group reported more URTIs and ear infections than the 4-weekly group and fewer LRTIs (Table 3). The rate of other infections per patient-year was 3.68 in the total patient group, with a higher rate for patients in the 3-weekly schedule (4.19) than in the 4-weekly schedule (3.33).

**Table 3:** Number of patients with other infections, rate of other infections per patient-year, main study population

Other infections	3-weekly IVIG schedule (*n* = 21)	4-weekly IVIG schedule (*n* = 30)	Total (*n* = 51)
Total no. of patients with other infections, *n* (%)	18 (85.7)	21 (70.0)	39 (76.5)
Ear infections	5 (23.8)	4 (13.3)	9 (17.6)
Eye infections	1 (4.8)	2 (6.7)	3 (5.9)
GI tract infections	7 (33.3)	10 (33.3)	17 (33.3)
Genitourinary tract infections	2 (9.5)	3 (10.0)	5 (9.8)
URTI	14 (66.7)	14 (46.7)	28 (54.9)
LRTI	4 (19.0)	9 (30.0)	13 (25.5)
Skin infections	2 (9.5)	2 (6.7)	4 (7.8)
Infections not classified elsewhere	4 (19.0)	6 (20.0)	10 (19.6)
Total number of other infections, *n*	86	99	185
Number of patient-year exposure	20.5	29.7	50.2
Rate of other infections per patient-year	4.19	3.33	3.68
One sided 95% CI, upper limit	6.89	5.17	5.12

*CI* confidence interval, *GI* gastrointestinal, *IVIG* intravenous immunoglobulin, *LRTI* lower respiratory tract infection, *URTI* upper respiratory tract infection

The mean time to resolution of infection was 14.3 days for SBIs and 18.4 days for other infections. The time to resolution of non-serious infections was higher in the 4-weekly group vs the 3-weekly group (21.4 vs 14.9 days), but high standard deviations were seen in both groups (data not shown).

Antibiotics were used by 82.4% of patients during the course of the study of which 86.0% were for therapeutic reasons, while the rest were prophylactic use. The percentage of patients using antibiotics was similar between treatment groups (data not shown).

Overall, 49.0% of patients in the study had a total number of 68 absences from work or school due to infections; the percentage of patients taking absences and the number of absences were higher in the 3-weekly group (13 of 21 patients [61.9%] and 37 absences) than in the 4-weekly group (12 of 30 patients [40.0%] and 31 absences). There were 183 days missed from work/school during the study, and the mean number of days missed per patient-year was 3.64.

Only one patient treated at 4-week intervals was hospitalized due to infections (for bacterial pneumonia; total duration of hospital stay 4 days; overall rate of days in hospital per patient-year 0.080). Fourteen episodes of fever occurred in 11 patients (Table 4).

**Table 4:** Total number of episodes of fever in the main study population

Episodes of fever^a^	Children ≥2 years, <12 years (*n* = 13)	Adolescents ≥12 years, <16 years (*n* = 12)	Adults ≥16 years, ≤75 years (*n* = 26)	Total (*n* = 51)
No. of patients with fever, *n* (%)	5 (38.5)	1 (8.3)	5 (19.2)	11 (21.6)
Total no. of episodes of fever, *n*	6	2	6	14
Rate of episodes of fever per patient-year	0.463	0.174	0.233	0.279

*no.* number

^a^As determined by the investigator

### Safety

#### Main Study

Of the 51 patients in the safety set, 48 (94.1%) experienced ≥1 AE during the study. No AEs led to study withdrawal or death. Serious (SAEs) and severe AEs occurred in five (9.8%) and seven patients (13.7%), respectively. No children had SAE, but four adults (15.4%) and one adolescent (8.3%) had seven SAEs, all considered unrelated (gout, pneumonia, bronchiectasis [twice], bronchospasm, septoplasty, and thrombocytopenia). Table 5 lists AEs experienced by >10% and related AEs experienced by >3% of treated patients. Patients in the 4-weekly group had a higher incidence of SAEs (13 vs 5%). In contrast, more patients in the 3-weekly treatment group had severe AEs than patients in the 4-weekly group (24 vs 7%).

**Table 5:** Display of all adverse events (AEs; frequency >10% of the 51 total patients) and study medication-related AEs (frequency >3%) by MedDRA System Organ Class and Preferred Term in the main study population

Adverse event	All AEs, *n* (%)	Related AEs, *n* (%)
Infections and infestations	40 (78.4)	0 (0.0)
Upper respiratory tract infection	15 (29.4)	
Nasopharyngitis	13 (25.5)	
Sinusitis	13 (25.5)	
Bronchitis	8 (15.7)	
Gastroenteritis	8 (15.7)	
Otitis media	7 (13.7)	
Influenza	6 (11.8)	
Pharyngitis	6 (11.8)	
Gastrointestinal disorders	27 (52.9)	7 (13.7)
Abdominal pain	11 (21.6)	5 (9.8)
Nausea	7 (13.7)	4 (7.8)
Vomiting	7 (13.7)	2 (3.9)
General disorders and administration site conditions	27 (52.9)	8 (15.7)
Pyrexia	11 (21.6)	3 (5.9)
Fatigue	10 (19.6)	2 (3.9)
Chills		2 (3.9)
Nervous system disorders	20 (39.2)	9 (17.6)
Headache	14 (27.5)	9 (17.6)
Respiratory, thoracic and mediastinal disorders	20 (39.2)	1 (2.0)
Cough	7 (13.7)	1 (2.0)
Musculoskeletal and connective tissue disorders	14 (27.5)	1 (2.0)
Pain in extremity	6 (11.8)	
Injury, poisoning and procedural complication	9 (17.6)	0 (0.0)
Skin and subcutaneous tissue disorders	9 (17.6)	0 (0.0)
Metabolism and nutrition disorders	6 (11.8)	0 (0.0)

During the course of this study, treatment-emergent AEs that were classified by the investigator to be related to the study medication occurred in 16 patients (31.4%); six patients (28.6%) enrolled in the 3-weekly treatment schedule and ten patients (33.3%) in the 4-weekly treatment schedule. The age distribution was as follows: 42.3% of adults (62.5% 3-weekly; 33.3% 4-weekly group), 25.0% of adolescents (12.5% 3-weekly; 50.0% 4-weekly group), and 15.4% of children (none 3-weekly; 25.0% 4-weekly group). The most common events in adults were headache (26.9%), nausea (11.5%), and vomiting, upper abdominal pain, and pyrexia (7.7% each), while in adolescents, the most common were headache, pyrexia, fatigue, and chills (8.3% each) and in children abdominal pain (15.4%) and chills, headache, nausea, and ear pain each in one case (7.7%). Only two patients received premedication (3.9%) for three infusions (0.4%). The maximum infusion rate of 0.08 mL/kg/min was used in 90.1% of infusions after the seventh infusion. Study medication-related (possible or probable) treatment-emergent AEs occurred during 38 infusions (5.1%: 2.7% in children, 2.2% in adolescent, and 7.8% in adult infusions). Study medication-related headache was the most abundant and noted in 22 infusions (3.0%). Most of these (35/38) occurred within 72 h after end of infusion.

Only 13 infusions had clinically significant abnormal values in hematology parameters or urinalysis, and none was present in three or more infusions. There were no abnormalities in direct Coombs test, biochemical assessments, viral markers, vital signs, and physical examinations. Three patients had hematological AEs; one patient had leukopenia (no treatment was required), one had thrombocytopenia, and one had anemia (both resolved due to effective treatment). No change to the study medication administration schedule was required in these patients.

#### Extension Study

Of the 21 patients in the safety set, 17 (81.0%) experienced at least one AE during the study which were generally mild to moderate in intensity with only one patient having severe AEs (4.8%). No AEs led to study withdrawal or death. No SAEs were reported. Table 6 lists AEs experienced by at least two (9.5%) and the related AEs experienced by >3% of treated patients. There was a higher overall incidence of AEs in patients receiving the 3-week treatment schedule than the 4-week schedule (91.7 vs 66.7%), as well as a higher incidence of related AEs (25.0 vs 11.1%) and severe AEs (8.3 vs 0%).

**Table 6:** Display of all adverse events (AEs) and study medication-related AEs (frequency at least two [all AEs] or one [related AEs] of the total 21 patients) by MedDRA System Organ Class and Preferred Term in the extension study population

Adverse event	All AEs, *n* (%)	Related AEs, *n* (%)
Infections and infestations	9 (42.9)	0 (0.0)
Sinusitis	4 (19.0)	
Nasopharyngitis	2 (9.5)	
Gastrointestinal disorders	7 (33.3)	3 (14.3)
Nausea	3 (14.3)	2 (9.5)
Vomiting	3 (14.3)	0 (0.0)
Abdominal pain	2 (9.5)	1 (4.8)
Diarrhea	2 (9.5)	0 (0.0)
General disorders and administration site conditions		1 (4.8)
Chest pain	4 (19.0)	1 (4.8)
Pyrexia	2 (9.5)	0 (0.0)
Nervous system disorders	2 (9.5)	2 (9.5)
Headache	2 (9.5)	2 (9.5)
Respiratory, thoracic and mediastinal disorders	5 (23.8)	0 (0.0)
Musculoskeletal and connective tissue disorders	2 (9.5)	2 (9.5)
Arthralgia	<2	1 (4.8)
Musculoskeletal pain	<2	1 (4.8)
Injury, poisoning and procedural complication	6 (28.6)	1 (4.8)
Contusion	2 (9.5)	0 (0.0)
Vascular procedure complication	<2	1 (4.8)
Skin and subcutaneous tissue disorders	2 (9.5)	0 (0.0)

AEs were considered treatment-related in four patients (19.0%); two children had a total of three related AEs (abdominal pain, headache, and vascular procedure complication each 12.5%) and two adults had a total of six related AEs (nausea 20%; arthralgia, musculoskeletal pain, headache, and chest pain, each 10%).

No patients received premedication. The maximum infusion rate of 0.14 mL/kg/min was used in 19/21 patients. Study medication-related (possible or probable) treatment-emergent AEs were observed with six infusions (6.3%), 8.3% in children, none in adolescent, and 6.7% in adult infusions. Headache was the most abundant event during the extension study and noted in three infusions (3.1%).

There were no clinically significant changes in laboratory parameters and no prominent results from vital signs or physical examination during the extension period.

## Discussion

Both the FDA and EMA recommend that a finding of a serious infection rate per patient-year of <1.0 is adequate evidence of efficacy of IVIG as substitution therapy [8, 9]. The current study meets this requirement, with a rate of SBI as low as 0.08 overall, confirming the efficacy of panzyga® in preventing the occurrence of SBIs in patients with PID. This low rate of SBIs as well as the low rate of other infections (3.7 per patient/year) further confirms that the dosing and corresponding trough levels observed in this study were adequate. The average dose calculated by body weight at each infusion was 485 mg/kg body weight, which is in line with doses recommended in the core summary of product characteristics. Serum IgG trough levels were nearly constant for both treatment schedules during the course of the study and exceeded the trough level of 600 mg/dL recommended by EMA [10].

The results from the present study are consistent with other clinical trials investigating the efficacy of IVIG in patients with antibody-deficient PID. A study of 80 adults and children with CVID or XLA treated with Privigen® 10% at a dose of 200–888 mg/kg every 3 or 4 weeks for 12 months had an annual SBI rate of 0.08 (upper one-sided 97.5% CI 0.182), while the annual rate of all infections was 3.55 [11], similar rates to those seen in the present study. The average annual rate of missed school or work days was 7.94 days/patient [11], a higher rate than what was seen with panzyga®. In another study of 22 patients with PID receiving 300–450 mg/kg Kiovig 10% every 3 weeks, no episodes of severe infection were reported, and the median monthly rate of mild or moderate infection episodes was 0.48. The rate of days off work/school per month ranged from 0 to 1.58 in the observational period [12].

Other clinical trials include a 46-patient study of octagam® 5% IVIG (400 or 600 mg/kg every 28 days or 300–450 mg/kg every 21 days) for 12 months, in which the estimated infection rate was 0.1 SBIs/patient/year (98% CI 0.033–0.279) and mean number of days of work or school missed was 5 during the course of the study; both outcomes were similar to those in the present study [13]. A study with Flebogamma® 10% DIF infused at a dose of 300–600 mg/kg every 3 or 4 weeks for 12 months in 46 PID patients reported that the overall rate of acute SBIs/patient/year was 0.025 (98% CI 0.001–0.133) and the overall mean rate of all infections was 2.2/patient/year, with 43% patients reporting missing at least 1 day of work/school/usual activities, with the mean number of days lost being 3.0 [14]. A 12-month study of Bivigam® 300–800 mg/kg infused every 3 or 4 weeks into 63 patients with PID demonstrated a SBI rate of 0.035/patient/year, and a general infection rate of 2.6/patient/year. Days off work or school were 2.28/patient/year [15], results again similar to our study.

In the present study, the proportion of patients who experienced infections other than SBIs was greater and the rate of URTIs was higher in the 3-weekly group than in the 4-weekly group. This could have been a chance occurrence that may have taken place in any random grouping of patients, as the patients continued with their treatment schedule and dose that they had received before enrolment into this study. Alternatively, it is possible that patients who were placed on a 3-weekly schedule for IVIG treatment were likely to have a more compromised immune system and, therefore, be at greater risk of developing such infections.

The majority of AEs were assessed as mild; none led to study withdrawal or death. None of the patients in the study exhibited signs of hemolysis. As expected, the most frequent related AE observed was headache. The tolerability and safety profile was excellent at high infusion rates. Indeed, panzyga® could be safely administered at infusion speeds that were equal [16] or considerably higher than used in most clinical trials treating antibody-deficient patients with IVIG, exceeding the maximum approved rates for most IVIG preparations [11, 17, 18]. Most patients (>90%) in the extension study tolerated the highest infusion speed of 0.14 mL/kg/min (840 mg/kg/h), without any associated increase in rates of AEs. In fact, there was a decrease in the related AE rate from 31.4% in the main study to 19.0% in the extension study.

The strengths of the study include its multicenter, international design, and the wide age range of the patients, which makes the results applicable to an extended population. The high proportion of patients (96%) completing the study without any major protocol violation is an additional strength. Limitations of the study include the lack of a comparator group and a lack of blinding, although it should be noted that this study was designed in accordance with the study design recommendations of the FDA and the EMA for studies of IVIG in the treatment of PID [8, 9].

## Conclusion

These results demonstrate that treatment with panzyga® is highly effective in PID patients with predominant antibody deficiency and has excellent tolerability; patients exhibited a very low level of SBIs and a low rate of related AEs, even when infusion rates were increased up to 0.14 mL/kg/min.
